# Prevalence of antimicrobial drug resistant bacteria carried by in- and outpatients attending a secondary care hospital in Zambia

**DOI:** 10.1186/s13104-017-2710-x

**Published:** 2017-08-10

**Authors:** Marjolijn M. B. Nagelkerke, Kapembwa Sikwewa, Dennis Makowa, Irene de Vries, Simon Chisi, J. Wendelien Dorigo-Zetsma

**Affiliations:** 1 Saint Francis’ Hospital, Katete, Zambia; 2 Department of Medical Microbiology, Tergooi Hospital, Hilversum, The Netherlands

**Keywords:** Antimicrobial resistance, Antimicrobial susceptibility testing, Antibiotic stewardship, Community carriership, Hospitalization, MRSA, Enterobacteriaceae, ESBL, Africa

## Abstract

**Objective:**

Antimicrobial resistance is an increasing global health problem. Very little data on resistance patterns of pathogenic bacteria in low-income countries exist. The aim of this study was to measure the prevalence of antimicrobial drug resistant bacteria carried by in- and outpatients in the resource constraint setting of a secondary care hospital in Zambia. Nasal and rectal samples from 50 in- and 50 outpatients were collected. Patients were randomly selected and informed consent was obtained. Nasal samples were tested for the presence of methicillin-resistant *Staphylococcus aureus* (MRSA), and rectal samples for Gram-negative rods (family of Enterobacteriaceae) non-susceptible to gentamicin, ciprofloxacin and ceftriaxone. Additionally, E-tests were performed on ceftriaxone-resistant Enterobacteriaceae to detect extended-spectrum β-lactamases (ESBLs).

**Results:**

14% of inpatients carried *S. aureus*, and 18% of outpatients. No MRSA was found. 90% of inpatients and 48% of outpatients carried one or more Enterobacteriaceae strains (75% *Escherichia coli* and *Klebsiella pneumonia*) resistant to gentamicin, ciprofloxacin and/or ceftriaxone (p < 0.001). Among inpatients gentamicin resistance was most prevalent (in 78%), whereas among outpatients ciprofloxacin resistance prevailed (in 38%). All ceftriaxone-resistant Enterobacteriaceae were ESBL-positive; these were present in 52% of inpatients versus 12% of outpatients (p < 0.001). We conclude it is feasible to perform basic microbiological procedures in the hospital laboratory in a low-income country and generate data on antimicrobial susceptibility. The high prevalence of antimicrobial drug resistant Enterobacteriaceae carried by in- and outpatients is worrisome. In order to slow down antimicrobial resistance, surveillance data on local susceptibility patterns of bacteria are a prerequisite to generate guidelines for antimicrobial therapy, to guide in individual patient treatment and to support implementation of infection control measures in a hospital.

**Electronic supplementary material:**

The online version of this article (doi:10.1186/s13104-017-2710-x) contains supplementary material, which is available to authorized users.

## Introduction

Antimicrobial resistance (AMR) amongst bacteria is a growing worldwide problem [1, 2]. In a recent United Nations Meeting on antimicrobial resistance [3], low and middle income countries were predicted to have the greatest problems in managing drug resistance and the subsequent burden of disease. Yet, scant data exist on current resistance patterns of common pathogenic bacteria in these countries [4]. Also, the treatment of bacterial infections in individual patients is largely empirical, due to low access to diagnostic microbiological laboratories. The lack of surveillance data and feedback from clinical microbiology laboratories on etiology and susceptibility patterns of bacterial infections in individual patients hinders antibiotic stewardship and appropriate antibiotic use [5]. Apart from being useful for patient treatment, data on circulating drug resistant bacteria can emphasize the necessity of rigorous infection control measures and hygiene practices. Adequate infection control plays an important role in reducing both the burden of infection and the spread of resistant microorganisms.

Data on resistance patterns of bacteria from the sub-Saharan African region mainly focus on clinical isolates from hospitalized patients [6]. To gain insight in the prevalence of antimicrobial drug resistant bacteria in the population, we performed a prospective study on colonization rate with drug resistant bacteria among patients attending the outpatient department (OPD) of a secondary care hospital in Eastern Zambia. In addition, we compared the colonization rates of AMR bacteria among the outpatients with that of hospitalized patients to assess the influence of hospitalization on colonization with AMR bacteria.

## Methods

### Study participants

A prospective point-prevalence study was conducted during 3 months in 2015/6 in a secondary care hospital (350 beds, serves a population of ~1.5 million) in the rural area of the Eastern Province in Zambia.

Adult study participants (≥18 years) were selected randomly from in- and outpatient departments. All participants were properly informed and signed an informed consent. Nasal and rectal samples from inpatients (medical and surgical wards; inclusion criterion: hospital stay >48 h at inclusion) and outpatients (exclusion criterion: hospitalization or antibiotic treatment 2 weeks prior to inclusion) were collected. For each participant data on gender, age, antibiotic use and length of hospital stay (inpatients) were collected and anonymized upon entering in a database. 100 patients (50 out- and 50 inpatients) was estimated to be an adequate sample size, without calculations, as no prevalence data on colonization rate of AMR bacteria for the region exist.

A one-way ANOVA was used to compare resistance percentages for the different antibiotics between the in- and outpatient group. p values were not adjusted for multiple comparisons; p values <0.05 were considered statistically significant. Statistical analysis was performed in SPSS 22.0 for Windows (IBM, Armonk, NY, USA).

### Microbiology procedures

Laboratory facilities are available at the hospital and basic microbiology procedures can be performed. The study focused on methicillin-resistant *S. aureus* (MRSA) from nasal swabs, as (carriage of) MRSA is a global health concern. To screen for carriage of resistant gut flora Enterobacteriaceae, isolated from rectal swabs, were tested for susceptibility to gentamicin, ciprofloxacin and ceftriaxone. These antibiotics represent three different antibiotic classes (see Table 1), all with bactericidal activity. The selected antibiotics are key drugs in the treatment of severely ill patients (see hospital guidelines for treatment with the tested antibiotics, Table 1).

**Table 1:** Antibiotic class and hospital guidelines for treatment with cloxacillin, gentamicin, ciprofloxacin, and ceftriaxone

	Antibiotic class	First-line treatment for	Second-line treatment for
Cloxacillin	Penicillins	–	Skin infections
			Cellulitis
Gentamicin	Aminoglycosides	(Neonatal) sepsis	–
		Neonatal meningitis	
		Pyelonephritis (together with ciprofloxacin)	
Ciprofloxacin	Fluoroquinolones	Pyelonephritis (together with gentamicin)	Simple urinary tract infection
		Bloody diarrhea	
Ceftriaxone	Third-generation cephalosporins	–	Sepsis
			(Neonatal) meningitis
			Severe pneumonia
			Pyelonephritis

Nasal swabs were inoculated on a blood agar plate (10% human blood in Columbia Blood Agar Base solution) on the day of collection and incubated overnight at 37 °C. Next day *S. aureus* suspected colonies were identified using Gram stain, catalase, tube coagulase and DNase test. Susceptibility to oxacillin was tested on a Mueller–Hinton agar plate, using agar disk diffusion method (Kirby–Bauer) with oxacillin disk (1 μg) (Neo-Sensitabs tablets, Rosco Diagnostica, Taastrup, Denmark) and zones were read after inoculation (24 h) [7].

Rectal swabs were inoculated on a blood agar plate, covering the whole surface resulting in semi-confluent growth. Antibiotic disks (gentamicin 10 μg, ciprofloxacin 5 μg, ceftriaxone 30 μg) (Neo-Sensitabs tablets, Rosco Diagnostica, Taastrup, Denmark) were added directly to the inoculated plate and plates were incubated overnight at 37 °C. Next day colonies present within the antibiotic disks’ inhibition zone were picked and Gram stained. Gram-negative, rod shaped bacteria were tested for oxidase. Antimicrobial susceptibility testing (AST) for gentamicin, ciprofloxacin and ceftriaxone of the isolated oxidase-negative, Gram-negative rods was confirmed, testing the pure culture with 0.5 McFarland inoculum by disk diffusion on Mueller–Hinton agar. Inhibition zones were interpreted according to Clinical and Laboratory Standards Institute (CLSI) guidelines [7]. Samples were shipped to the reference laboratory (Tergooi Hospital, The Netherlands) for further identification of the oxidase-negative Gram-negative rods (Maldi-tof MS Microflex LT SH System) and confirmation of extended-spectrum β-lactamase (ESBL) positivity in ceftriaxone-resistant isolates using Etest™ strips (AB BIODISC, Solna, Sweden) according to standard procedures [8].

## Results

100 patients were included (50 from the OPD and 50 from the hospital wards). 19 patients did not fulfill the inclusion criteria and were excluded (5 were <18 years, 8 inpatients had been hospitalized <48 h, 2 outpatients had been using antibiotics the prior 2 weeks and 4 patients refused to participate). Patient characteristics are specified in Additional file 1: Table S1.

### Nasal samples

*Staphylococcus aureus* was isolated from the nasal swabs of 16 patients. There was no significant difference (p = 0.590) in *S. aureus* carriage between in- and outpatients (respectively 14 and 18% carriage). None of the *S. aureus* strains was resistant to oxacillin. Therefore, no patient was found to carry MRSA.

### Rectal samples

**Fig. 1:**
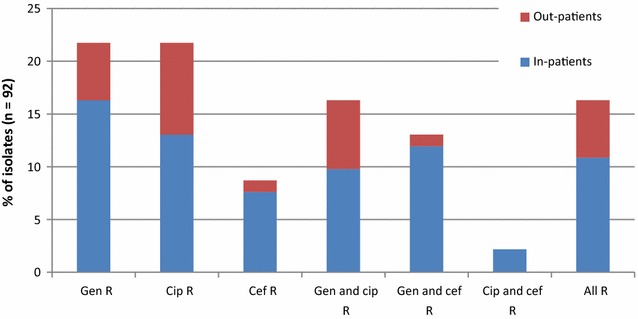
Distribution of antibiotic resistance among 92 isolates. *Gen* gentamicin, *Cip* ciprofloxacin, *Cef* ceftriaxone. Gen R indicates an isolate was only resistant to gentamicin. Gen and cip R indicates an isolate was resistant to both gentamicin and ciprofloxacin, but not ceftriaxone. All R indicates an isolate was resistant to the three antibiotics tested

92 oxidase-negative Gram-negative rods that were resistant to one or more of the antibiotics gentamicin, ciprofloxacin and ceftriaxone were isolated from 69 patients. In Table 2 the results are specified per antibiotic and for all antibiotics combined. The number of patients carrying an isolate resistant to an antibiotic and to the antibiotics combined was significantly higher among inpatients compared to outpatients. Note that some isolates were resistant to multiple antibiotics. The distribution of antibiotic resistance among the 92 isolates is shown in Fig. 1.

**Table 2:** Prevalence of resistant Enterobacteriaceae in rectal samples among inpatients and outpatients

	Inpatients (n = 50)	Outpatients (n = 50)	*p* value
Total			
# of resistant isolates	66	26	<0.001
# of patients carrying resistant isolates (%) [95% confidence interval]	45 (90%) [81, 99]	24 (48%) [34, 62]	
Gentamicin			
# of resistant isolates	45	17	<0.001
# of patients carrying resistant isolates (%) [95% confidence interval]	39 (78%) [66, 90]	16 (32%) [19, 45]	
Ciprofloxacin			
# of resistant isolates	33	19	0.028
# of patients carrying resistant isolates (%) [95% confidence interval]	30 (60%) [46, 74]	19 (38%) [24, 52]	
Ceftriaxone (all ESBL positive)			
# of resistant isolates	30	7	<0.001
# of patients carrying resistant isolates (%) [95% confidence interval]	26 (52%) [38, 66]	6 (12%) [3, 21]

Additional identification of the resistant isolates revealed that *Escherichia coli* was the most predominant species (56.5%), followed by *Klebsiella pneumoniae* (18.5%) and other species (25.0%) (Additional file 2: Figure S2). All ceftriaxone-resistant isolates were confirmed ESBL-positive by Etests.

## Discussion

In the hospital region antibiotics are easily obtainable, both for free when prescribed in rural health clinics, and for sale without prescription in drug stores. In the resource constraint setting of the hospital, it was feasible to perform a prevalence study on colonization with AMR bacteria among in- and outpatients. We focused on colonization with MRSA and drug resistant Enterobacteriaceae. For *S. aureus* a low colonization rate of 18 and 14% (out- respectively inpatients) in nasal samples was detected and no MRSA was detected. The relatively low nasal colonization rate of *S. aureus* was surprising, compared to an average *S. aureus* colonization rate of 30% in developed countries [9]. However, similar percentages have been reported for sub-Saharan African countries before [10]. The fact that we found no MRSA was also unexpected, as in different regions in sub-Saharan Africa MRSA percentages among *S. aureus* strains have been reported to vary between 8 and 13.4% [11], 19% [2] and 1–15% [12]. However, these were clinical MRSA strains, isolated from wounds or patients’ blood. Although we found no indications for MRSA carriership, it would still be interesting to investigate *S. aureus* isolates from infectious sites in patients for susceptibility to oxacillin.

The rectal colonization rate with drug resistant Enterobacteriaceae (mainly *E. coli* and *K. pneumoniae*) was high. 90% of inpatients and 48% of outpatients carried Enterobacteriaceae strains resistant to one or more of the antibiotics gentamicin, ciprofloxacin, and ceftriaxone.

The high colonization rate with gentamicin-resistant Enterobacteriaceae both among in- and outpatients (78 and 32% respectively) is alarming, as the hospital uses gentamicin as first-line treatment in patients with sepsis, neonatal meningitis and pyelonephritis. Although this resistance was detected in screening isolates, and not clinical isolates, it has been shown that asymptomatic carriers are often colonized with resistant bacteria that subsequently lead to infection [13]. Gentamicin resistance among Enterobacteriaceae in sub-Saharan African countries has been reported to vary up to 36% [2] and 47% [11] for *Klebsiella*, 29% [2] and 35% [11] for *E. coli*, and 25% (urban) to 4.7% (rural) [14] for both *Klebsiella* and *E. coli*, depending, amongst others, on the species and whether studies were performed in rural or urban areas. Considering these resistance percentages, gentamicin as an affordable first-line antibiotic for severe infections will likely become less efficacious in clinical treatment, leaving third-generation cephalosporins and fluoroquinolones as drugs of choice for empirical treatment.

Additionally, as may have been facilitated by increased consumption of these drugs, we detected a high colonization rate with ceftriaxone- and ciprofloxacin-resistant Enterobacteriaceae. All ceftriaxone-resistant isolates appeared to be ESBL-producers, making this third-generation cephalosporin a surrogate marker for ESBL-positivity [15], and ceftriaxone-resistant isolates non-susceptible for all β-lactam antibiotics. Ciprofloxacin (available for oral and intravenous treatment) resistance was high among both outpatients and inpatients. Possibly, the oral formulation and thus availability for outpatient treatment of ciprofloxacin is responsible for the high ciprofloxacin resistance among outpatients, compared to gentamicin and ceftriaxone. High ciprofloxacin resistance percentages for Enterobacteriaceae isolated from outpatients and health clinic attenders in sub-Saharan Africa have been reported [14]. However, it is well known that persons who have never been treated with antibiotics such as gentamicin or ceftriaxone can carry strains resistant to these antibiotics, apparently acquiring those from the environment [16]. In the outpatients we studied, this might be the case, as they had not been admitted to the hospital nor been treated with antibiotics at least 2 weeks before they were sampled.

We found a significant difference in colonization rate with resistant Enterobacteriaceae between in- and outpatients, with a higher percentage of inpatients carrying resistant strains. During the time inpatients were hospitalized their intestinal microbiome could have changed. Several factors have been reported to play a role in the change of the gastrointestinal flora during hospital admission, such as the underlying disease, antibiotic treatment and acquisition of (multi-resistant) microorganisms from the environment during the stay [17]. The latter can be facilitated under poor hygienic circumstances and clonal spread of resistant bacteria. For infections, the role of multidrug-resistant bacteria acquired during hospital admission, so called health care associated infections (HAI), has been extensively described [18, 19]. Given the difference in colonization rates with resistant bacteria in our OPD- and hospitalized patients, it would be interesting to further study resistance patterns of clinical isolates in these patient groups.

## Conclusions

We experienced it feasible to perform antibiotic susceptibility testing in the hospital laboratory in a low-income country, and found a high colonization rate with drug resistant bacteria among in- and outpatients. To slow down the increase of AMR, interventions like antibiotic stewardship and infection control procedures have been proposed [20]. The availability of data on susceptibility of locally circulating bacterial strains will support health care workers to perform these interventions.

## Limitations

Microbiology procedures in the laboratory were performed under basic circumstances, and no quality control program for AST was implemented. Resistant Enterobacteriaceae were cultured from blood agar plates with antibiotic disks, which is not a standard procedure. The phenotypic detection of ESBL-production was not followed by genetic analysis for the existence of resistance genes. Only a limited number of antibiotics was tested, with commonly used antibiotics like amoxicillin (clavulanate), sulphamethoxazole/trimethoprim or chloramphenicol missing. However, the study was set up to generate information on carriage of bacteria resistant to key antibiotics, used in critically ill patients. Furthermore, the tested antibiotics for Enterobacteriaceae represent three different antibacterial drug classes and especially bacteria with resistance to ≥3 antibacterial drug classes are defined as multidrug-resistant and have become a cause for serious concern [21]. Finally, the patients attending the OPD are not representative for the general population. Although not having consumed antibiotics nor being admitted to a hospital in the weeks before inclusion in the study, they were suffering an illness and could have been attending health centers elsewhere. It is possible that colonization rates of resistant bacteria in people from the community will be lower than we found in our study population.

## Additional files

**Additional file 1: Table S1.** Patient characteristics. The table contains information on patient average age, gender, antibiotic use and length of stay in hospital.

**Additional file 2: Figure S1.** Species identification of the resistant Enterobacteriaceae and their distribution among in- and outpatients. The figure specifies the species the resistant Enterobacteriaceae belong to and how these are distributed between the in- and outpatient group.

## Abbreviations

AMR: antimicrobial resistance
AST: antimicrobial susceptibility testing
CLSI: Clinical and Laboratory Standards Institute
ESBL: extended-spectrum β-lactamase
HAI: health care associated infections
MRSA: methicillin-resistant *Staphylococcus aureus*
OPD: outpatient department
