# Supplementary material for: Prevalence of antimicrobial drug resistant bacteria carried by in- and outpatients attending a secondary care hospital in Zambia
Source: BMC Res Notes. 2017 Aug 10;10:378. doi: 10.1186/s13104-017-2710-x (PMC5553783; doi:10.1186/s13104-017-2710-x)
Supplement: Supplementary file 1 — Additional file 1: Table S1. Patient characteristics. The table contains information on patient average age, gender, antibiotic use and length of stay in hospital. [file 13104_2017_2710_MOESM1_ESM.docx]

Table S1

Patient characteristics.

|  | **Average age** | **# of Women** | **# of patients received antibiotics past 2 weeks** | **Median number of days in hospital** |
| --- | --- | --- | --- | --- |
| **Inpatients (n=50)** | 37 | 29 | 41 | 5 |
| **Outpatients (n=50)** | 41 | 39 | N/A | N/A |

N/A: not applicable
