# Supplementary material for: Prevalence of antimicrobial drug resistant bacteria carried by in- and outpatients attending a secondary care hospital in Zambia
Source: BMC Res Notes. 2017 Aug 10;10:378. doi: 10.1186/s13104-017-2710-x (PMC5553783; doi:10.1186/s13104-017-2710-x)
Supplement: Supplementary file 2 — Additional file 2: Figure S1. Species identification of the resistant Enterobacteriaceae and their distribution among in- and outpatients. The figure specifies the species the resistant Enterobacteriaceae belong to and how these are distributed between the in- and outpatient group. [file 13104_2017_2710_MOESM2_ESM.docx]

Figure S1

Species identification of the resistant Enterobacteriaceae and their distribution among in- and outpatients
